# Albumin Nano-Encapsulation of Piceatannol Enhances Its Anticancer Potential in Colon Cancer Via Downregulation of Nuclear p65 and HIF-1α

**DOI:** 10.3390/cancers12010113

**Published:** 2020-01-01

**Authors:** Alaa A. A. Aljabali, Hamid A. Bakshi, Faruck L. Hakkim, Yusuf A. Haggag, Khalid M. Al-Batanyeh, Mazhar S. Al Zoubi, Bahaa Al-Trad, Mohamed M. Nasef, Saurabh Satija, Meenu Mehta, Kavita Pabreja, Vijay Mishra, Mohammed Khan, Salem Abobaker, Ibrahim M. Azzouz, Harish Dureja, Ritesh M. Pabari, Ashref Ali K. Dardouri, Prashant Kesharwani, Gaurav Gupta, Shakti Dhar Shukla, Parteek Prasher, Nitin B. Charbe, Poonam Negi, Deepak N. Kapoor, Dinesh Kumar Chellappan, Mateus Webba da Silva, Paul Thompson, Kamal Dua, Paul McCarron, Murtaza M. Tambuwala

**Affiliations:** 1 Department of Pharmaceutical Sciences, Yarmouk University—Faculty of Pharmacy, Irbid 566, Jordan; 2 School of Pharmacy and Pharmaceutical Science, Ulster University, Coleraine BT52 1SA, Northern Ireland, UK; 3 Department of Mathematics and Sciences, College of Arts and Applied Sciences Dhofar University Salalah, Salalah 211, Oman; 4 Department of Pharmaceutical Technology, Faculty of Pharmacy, University of Tanta, Tanta 31111, Egypt; 5 Department of Biological Sciences, Yarmouk University—Faculty of Science, Irbid 566, Jordan; 6 Department of Basic Medical Sciences, Yarmouk University—Faculty of Medicine, Irbid 566, Jordan; 7 Department of Pharmacy and Biomedical Sciences, School of Applied Sciences, University of Huddersfield, Queensgate, Huddersfield HD1 3DH, UK; 8 School of Pharmaceutical Sciences, Lovely Professional University, Phagwara, Punjab 144411, India; 9 Department of Gynecology, European Competence Center for Ovarian Cancer, Campus Virchow, Klinikum Charite-Universitatmedizin Berlin, augustenburger Platz 1, 13353 Berlin, Germany; 10 Department of Dermatology, Venerology, and allergology, Charite—Universitatsmedizin Berlin, Corporate Member of Freie Universitat Berlin, Chariteplatz1, 10117 Berlin, Germany; 11 Department of Pharmaceutical Sciences, Maharshi Dayanand University, Rohtak 124001, India; 12 School of Pharmacy, Royal College of Surgeons in Ireland, Dublin-09 D02 YN77, Ireland; 13 Department of Forensic Science, School of Applied Science, Huddersfield University, Queensgate, Huddersfield HD1 3DH, UK; 14 Department of Pharmaceutics, School of Pharmaceutical Education and Research, Jamia Hamdard, New Delhi 110062, India; 15 School of Pharmacy, Suresh Gyan Vihar University, Jagatpura, Jaipur 302017, India; 16 Priority Research Centre for Healthy Lungs, Hunter Medical Research Institute (HMRI) and School of Biomedical Sciences and Pharmacy, University of Newcastle, Callaghan, NSW 230, Australia; 17 Department of Chemistry, University of Petroleum & Energy Studies, Dehradun 248007, India; 18 Departamento de Química Orgánica, Facultad de Química y de Farmacia, Pontificia Universidad Católica de Chile, Av. Vicuña McKenna 4860, 7820436, Macul, Santiago 4860, Chile; 19 School of Pharmaceutical Sciences, Shoolini University of Biotechnology and Management Sciences, Solan, India 173229, India; 20 Department of Life Sciences, School of Pharmacy, International Medical University, Bukit Jalil, Kuala Lumpur 57000, Malaysia; 21 School of Biomedical Sciences, University of Ulster, Coleraine BT52 1SA, UK; 22 Discipline of Pharmacy, Graduate School of Health, University of Technology, Sydney, NSW 2007, Australia; 23 Centre for Inflammation, Centenary Institute, Sydney, NSW 2050, Australia

**Keywords:** piceatannol, colon cancer, albumin nanoparticles, nuclear P65, HIF-1α

## Abstract

Piceatannol (PIC) is known to have anticancer activity, which has been attributed to its ability to block the proliferation of cancer cells via suppression of the NF-kB signaling pathway. However, its effect on hypoxia-inducible factor (HIF) is not well known in cancer. In this study, PIC was loaded into bovine serum albumin (BSA) by desolvation method as PIC–BSA nanoparticles (NPs). These PIC–BSA nanoparticles were assessed for in vitro cytotoxicity, migration, invasion, and colony formation studies and levels of p65 and HIF-1α. Our results indicate that PIC–BSA NPs were more effective in downregulating the expression of nuclear p65 and HIF-1α in colon cancer cells as compared to free PIC. We also observed a significant reduction in inflammation induced by chemical colitis in mice by PIC–BSA NPs. Furthermore, a significant reduction in tumor size and number of colon tumors was also observed in the murine model of colitis-associated colorectal cancer, when treated with PIC–BSA NPs as compared to free PIC. The overall results indicate that PIC, when formulated as PIC–BSA NPs, enhances its therapeutic potential. Our work could prompt further research in using natural anticancer agents as nanoparticels with possible human clinical trails. This could lead to the development of a new line of safe and effective therapeutics for cancer patients.

## 1. Introduction

Colorectal cancer (CRC) is the third most common cancer in men and the second in women worldwide, with a prevalence of 10.0 and 9.2%, respectively [1,2,3]. CRC is the fourth most common cancer in the United Kingdom (UK), accounting for 12% of all new cancer cases [4] Despite the advances in our knowledge in managing CRC, current treatments are not efficient to control metastatic forms of CRC [5]. Surgery is the main procedure in stage I patients with potentially curable CRC, but neoadjuvant chemotherapy and/or radiotherapy is sometimes given before or after surgery depending on the disease stage. However, these treatment regimens are not sufficient to control the progressing CRC, since 30% of patients with stage I–III and up to 65% of patients with stage IV will develop recurrent disease [6]. This highlights the need for finding new and more effective treatment schemes and strategies.

Natural products are a potential source for the development of effective and safe novel anticancer molecules. The potential of nutraceutical natural compounds such as flavonoids, polyphenols, anthocyanins, carotenoids, or terpenoids for cancer prevention has been widely investigated, and there are many pieces of evidence supporting that moderate consumption of fruits and vegetables is correlated with decreased risk of CRC [7]. Some members of these families of compounds can modulate signaling pathways, as well as regulate the expression of genes involved in cell cycle regulation, differentiation, and apoptosis [8]. Chronic mucosal inflammation is the most common factor involved in both ulcerative colitis and colorectal cancer [9,10]. Overexpression of pro-inflammatory cytokines is responsible for the induction of transcription factor nuclear factor kappa beta (NF-κβ) and hypoxia-inducible factor-1α (HIF-1α) [11,12]. Furthermore, overactivation of NF-κβ and HIF-1α leads to proliferation, migration, and invasion at the tumor site [13,14,15]. It is well documented that pro-inflammatory cytokines induce aberrant activation of NF-κβ and HIF-1α in ulcerative colitis and colorectal cancer patients [16,17,18]. This would make NF-κβ and HIF-1α proteins potential targets for effective treatment of colorectal cancer.

Piceatannol (trans-3,4,3′,5′-tetrahydroxystilbene, also known as 3-hydroxyresveratrol or astringinine) is a naturallyoccurring polyphenol and an analogue of the cancer chemopreventivee agent resveratrol (trans-3,5,4′-trihydroxystilbene) [19]. Piceatannol has anticancer and anti-inflammatory properties [20,21]. In recent years, it has been shown that piceatannol (PIC) could inhibit proliferation, migration, and metastasis of diverse cancers, such as colon, breast, prostate, and bladder cancers [22,23,24,25]. Specifically, the inhibition potential of PIC against NF-κβ and HIF-1α has been reported in different cancer cell lines [26,27,28]. However, it has low solubility in water, and it is less absorbable in the intestine, which alters its therapeutic potential [29,30]. To overcome this problem, a better drug solubilization strategy is needed, and nanotechnology is a promising means to address this unmet need.

The Food and Drug Administration (FDA) has approved several advances in human serum albumin (HSA)-binding therapies over the past two decades, and many more are under active clinical investigation. These successful discoveries are important to a wide range of therapeutics, particularly for cancer therapy and diabetes treatment. Ultimately, design is often extracted from a new understanding of HSA chemistry, accompanied by an insightful application to address an evolving medical problem. For example, Abraxane, a paclitaxel albumin nanoparticle, has become a pioneer for drug delivery technology based on nanomedicine and albumin with annual sales of $850 million in 2014 [31,32,33].

Due to its safety, biocompatibility, and flexibility, albumin has attracted considerable attention in recent years as a substrate for construction of nanoparticles (NPs) [31,32,33]. Albumin-based nanoparticle carrier systems represent an appealing strategy because, due to the different drug binding sites in the albumin molecule, a significant amount of drug can be incorporated into the particle matrix [34]. Due to the huge defined primary albumin structure and the high content of charged amino acids (e.g., lysine), albumin-based nanoparticles may allow positive (e.g., ganciclovir) or negative (e.g., oligonucleotide) molecules to be adsorbed electrostatically without the need for other compounds [35,36]. Bovine serum albumin (BSA) is highly water-soluble and binds with drugs and inorganic substances in a non-covalent manner. Therefore, its composition is homologous to the HAS (human serum albumin) three-dimensional structure. The main difference is the number of tryptophanes. BSA has two tryptophanes, while HAS only has one. This difference is useful in its spectrofluorometric analysis because this amino acid is the main residue responsible for the protein’s intrinsic fluorescence [37,38,39]. Due to its biocompatibility, biodegradable, non-toxic, and non-immunogenic features, it has been proposed that BSA can offer a suitable nanocarrier system for drug delivery [40,41,42,43].

In the present study. we design and characterize PIC-loaded BSA nanoparticles and evaluate its morphology, zeta potential, encapsulation efficiency, and controlled drug release profile. The effect of PIC–BSA NPs on in vitro anti-colon cytotoxicity, migration, invasion, and colony formation is further evaluated.

## 2. Materials and Methods:

### 2.1. Reagents and Chemicals

Piceatannol (PIC) (Cat no: P1928, purity >98%, CAS: −10083−24−6) was purchased from Tokyo Chemical Industry. Dextran sodium sulphate (DSS) (Cat no: −160110, lot no: −M2356, MW: −36000−50000) was obtained from MP Bio medicals. Bovine serum albumin (BSA) (Cat no: −A2153, Lot no-049k1585), Glutaraldehyde solution (Cat no: G5882) was ordered from Sigma. Ethanol (HPLC grade, Ulster University) and all other reagents were of analytical grade or higher purity. Milli-Q water was used throughout the study.

### 2.2. Cell Culture

CaCo-2 and HT-29 (human colon cancer cell lines) were generously provided by Keith Thomas, Ulster University. These cells were maintained as monolayer cultures in Dulbecco’s Modified Eagle’s Medium–High Glucose (DMEM–Hi) medium (Gibco BRL, Grand Island, NY, USA) and McCoy’s media supplemented with 10% fetal bovine serum (Gibco BRL, Grand Island, NY, USA) and 1% penicillin–streptomycin (Gibco BRL, Grand Island, NY, USA) at 37 °C in a humidified atmosphere (5% CO_2_).

### 2.3. Preparation of PIC-Loaded Bovine Serum Albumin Nanoparticles

PIC-loaded albumin nanoparticle was optimized as shown in the Figure S1. NP was fabricated by desolvation method as previously reported [44] with slight modification [45]. Briefly, the drug was dissolved in ethanol. The drug solution then added to BSA solution dropwise under constant magnetic stirring, which led to the formation of coacervates. Crosslinking agent, glutaraldehyde, was added after co-acervate formation. These co-acervates were stirred overnight to remove the organic solvent. Later, the resultant colloidal suspension was centrifuged to remove the organic solvent and washed with ultra-purified water. After centrifugation, the albumin nanoparticles were freeze-dried and store at 4 °C (Figure S2).

### 2.4. Characterization of Particle Size and Zeta Potential

The particle size and distribution of nanoparticles was determined by using Zetasizer (Zetasizer 5000, Malvern Instruments, Malvern, UK) based on the dynamic light scattering principle technique. A sample of nanoparticle suspension (5 mg/mL) was vortexed for 5 min. An aliquot from this suspension was diluted by ultrapure water and utilized to measure particle size in triplicate. A Zetasizer was used to measure the zeta potential of nanoparticles; 0.001 M KCl solutions with an adjusted conductivity were used to prepare diluted nanoparticle suspension. The principle of electrophoretic mobility under an electric field was used to measure the zeta potential values [46]. The results are expressed as the mean ± standard deviation of three measurements.

### 2.5. NMR Spectra of Nanoparticles

NMR spectra were run on a 500 MHz Bruker AVANCE DRX instrument using a broadband probe equipped with a z-gradient coil (Bruker-Biospin, Freemont, CA, USA). All NMR samples were 600 µL and run in standard 5 mm NMR tubes at 25 °C. Pulse programs used were standard sequences taken from the Bruker XWINNMR pulse sequence library. NMR experiments were set up and processed generally using the parameters suggested in 200 and More NMR Experiments: A Practical Course. The pulse programs selected for the two-dimensional (2D) COSY, HMQC, and HMBC experiments employed z gradients for coherence pathway selection. ^1^ H and ^13^ C chemical shifts were calibrated relative to the solvents and TMS [47].

### 2.6. Determination of Entrapment Efficiency

The entrapment efficiency of drugs in nanoparticles was calculated by the indirect method to determine drugs content in the supernatant. The concentration of non-encapsulated PIC in the supernatant was measured by a UV spectrophotometer at 430 nm, as described previously [48]. Briefly, a standard plot of the drug was prepared under identical conditions. The amount of entrapped PIC inside the NP was calculated by the difference between the initial amount of drug added and the measured non-encapsulated drug remaining in the external aqueous phase after NP fabrication. All measurements were assayed in triplicate and the average of each sample was recorded as the percentage curcumin encapsulation efficiency.

(1) $$\mathrm{Entrapment}\mathrm{efficiency}\left(\mathrm{EE}\right)=\frac{\mathrm{Experimental}\mathrm{drug}\mathrm{loading}}{\mathrm{practical}\mathrm{drug}\mathrm{loading}}\times 100$$

### 2.7. Determination of Percentage Yield

Percentage yield was calculated as previously reported [49], where the weight of the nanoparticles was divided by the total weight of drug and polymer used in the formulation as described below in the equation [49].

(2) $$\mathrm{Percentage}\mathrm{yield}=\frac{\mathrm{Total}\mathrm{weight}\mathrm{of}\mathrm{the}\mathrm{nanoparticles}}{\mathrm{Weight}\mathrm{of}\mathrm{drug}+\mathrm{Weight}\mathrm{of}\mathrm{polymer}}\times 100$$

### 2.8. In Vitro Drug Release

The in vitro release of drug from NPs was carried out in phosphate-buffered saline (PBS; pH 7.4, containing 0.1% w/v Tween-80) as previously described [50]. Briefly, 5 mg of PIC-loaded albumin NP was placed in a dialysis membrane (17 kD) and suspended in 1 mL PBS solution. The sample pouches were incubated at a temperature of 37 °C and a speed of 100 rpm in a reciprocal shaker water bath. The release samples from dialysis membrane were taken at predetermined time intervals of 0 min, 15 min, 30 min, 1 h, 2 h, 3 h, 6 h, 12 h, 24 h, 48 h, 72 h, 96 h, 120 h, 144 h, and 168 h. The collected samples were centrifuged for 5 min at 10,000 rpm. Each experiment was performed in triplicate.

### 2.9. Determination of Water Solubility

The water solubility of nanoparticles was determined by dissolving NPs and an equivalent amount of free drug in distilled water. The mixtures were kept for stirring overnight and centrifuged at 13,450 rpm for 10 min. The absorbance of the supernatant was measured by UV-spectroscopy [51].

### 2.10. Scanning Electron Microscopy of Nanoparticles

Nanoparticles’ surface morphology was characterized by the aid of scanning electron microscopy (SEM) on an FEI Quanta 400 FEG (FEI Company, Hillsboro, OR, USA). A sample of NP was mounted on carbon tape and sputter-coated with gold under vacuum in an argon atmosphere before imaging under SEM.

### 2.11. Cellular Uptake of Nanoparticles

Cellular uptake of NP was performed as previously reported [52]. Briefly, by seeding 1 × 10^5^ of HT-29 and CaCo-2 cells on chamber slide in 6-well plates. After incubation for 24 h, cells were washed 3 times with PBS. Cells were treated with 200 µg/mL of NP, then incubated for 3 h. After the 3 h incubation time elapsed, cells were washed with PBS to remove the excess NP from the wells and fixed with 4% paraformaldehyde for 15 min. The nucleus of cells was stained with 5 µg DAPI in 1 mL normal media and incubated for 15 min. The fluorescence images of different groups were obtained from phase-contrast microscopy.

### 2.12. In Vitro Cytotoxicity Assay

Cytotoxicity assays were performed as previously reported [53], with minor modifications. HT-29 and CaCo-2 cells (5 × 10^4^ cells/well in 500 µL media) were seeded in 24-well plates. The following day, cells were treated with the free drug and an equivalent amount of nanoparticles in Optimem^®^ media. The cytotoxic effect of free drug and nanoparticles was determined every 24 h using MTT assay to assess the viability of cancer cells. The treated cells were washed with 500 µL PBS, and 500 µL of 15% MTT dye solution in complete media was added to each well. The plates were incubated at 37 °C and 5% CO_2_ for an additional 4 h. The supernatant was discarded and formazan crystals were solubilized by adding 500 µL of DMSO, and the solution was vigorously mixed to dissolve the precipitate. The color intensity was measured at 570 nm (reference wavelength 630 nm) in a microplate reader (Fluostar Omega, BMG Lab Tech GMBH, Germany). The anti-proliferative effect of the free drug and nanoparticle treatment was calculated as a percentage of cell growth with respect to the DMSO and blank NP controls. The absorbance of the untreated cells was set at 100%. All of the experiments were repeated three times.

### 2.13. Migration Assay

Migration assay was performed through wound scratch assay as previously described [54]. Briefly, HT-29 and CaCo-2 cells were seeded (7.5 × 10^5^/well) into each well of 6-well plates. The cells were pipetted drop-by-drop around the edge of each well and shaken gently. The plates were placed in an incubator to allow cells to grow to confluence. A wound was introduced to confluent cells by using the yellow pipette tip by carefully scraping the tip down the center of each well. Old media were removed and 2 mL of fresh media with PLGA, albumin NP, and an equivalent amount of free drug were transfected into respective wells. Medium containing equivalent amounts of blank PLGA and albumin NP was used as the control. A photograph for each wound at 0 h was then taken and recorded. A photograph was taken at the same time of day at 24, 48, and 72 h. To quantify the results from the photographs, ImageJ software was used to measure the width of the wound. The width of the wound was measured at 3 different points in the photograph and the average was recorded. The degree of wound closure over 72 h was calculated in of all the treatments and compared with control and blank NPs. All of the experiments were repeated three times.

### 2.14. Colony Formation Assay

The colony formation assay was performed as previously described [55]. Briefly, 500 cells of HT-29 and CaCo-2 cells were seeded in 2 mL media in 6-well plates and allowed 2 days to attach and initiate colony formation. These cells were treated with PLGA NPs, albumin NPs, and the same amount of free drugs suspended in Optimem^®^ media over a period of 7 days. The plates were washed twice with PBS, fixed in chilled methanol, stained with Crystal Violet, washed with water, and air-dried. The number of colonies was counted by using a magnifying lens. The percent of colony formation was calculated by dividing the number of colonies formed in treatment by the number of colonies formed in blank NPs.

### 2.15. Invasion Assay

An invasion assay was performed as previously reported [56]. This was conducted to investigate the effect of nanoparticles and free drug treatment on the ability of HT-29 and CaCo-2 cells to invade a Matrigel-coated membrane. A number of 8 µm membrane transwell inserts were inserted into the well of 12-well plate (BD Bioscience, San Jose, CA, USA). The membrane transwell was coated with Matrigel before seeding the cancer cells. Matrigel was placed on ice and left to thaw at 4 °C, then diluted 1:1 with water, and 135 µL of the solution was placed carefully into the required number of inserts. The inserts were shaken gently to ensure the Matrigel solution spread evenly on the membrane. The whole plates were left in the laminar flow hood to dry for 60 min. Then, 4 × 10^4^ cells were seeded into each insert in a suspension of 200 µL of serum-free media. Next, 100 µL containing nanoparticles of drug and equivalent amount of free drug were treated into corresponding wells. The same volume of 100 µL containing equivalent amounts of blank NPs was used as the control. Finally, 700 µL of DMEM media containing 10% FBS was added to each well to bath the outer membrane surface and act as a chemoattractant. The next day, to new 12-well plates, 500 µL of methanol was added to the corresponding number of wells used. The media in both chambers were removed and cotton wool scrubbers were used to clean off any remaining cells or Matrigel layer. The inserts were placed into methanol for 10 min for fixation. Once fixation was completed, the inserts were left to dry. Using a scalpel, the membrane was cut out and placed with the original outer surface facing upwards in wells of a new 12-well plate. Then, 250 µL of Crystal Violet was then added to each well to stain up the cells that invaded through the membrane pores. The plates were left to be shaken for 20 min. Afterwards, the Crystal Violet solution was pipetted out and the membranes were washed thoroughly in their wells with Milli-Q water. The membranes were left to air dry for 60 min. Then, 250 µL of 70% ethanol was added to each membrane in each well and shaken for 30 min. Finally, 200 µL was pipetted out from each well and placed into the wells of 96-well plate. Absorbance at 590 nm was measured using a microplate reader (Fluostar Omega, BMG Lab Tech GMBH, and Germany). The effect of the free drug and an equivalent amount of nanoparticle treatments was calculated as a percentage of cell invasions with respect to blank NP controls. The absorbance of the untreated cells was set at 100%. All of the experiments were repeated three times.

### 2.16. Immunostaining for Cells

Immunostaining of cells was performed as previously reported [57]. A sterile glass coverslip was placed in 6-well plates. HT-29 and CaCo-2 cell was seeded at a 1.9 × 10^5^ cell density in 6-well plates. The plates were incubated for 24 h. The next day, plates were washed with sterile PBS to remove the dead cells from each well. Then, 7.5 µg/mL of free drug and nanoparticles were transfected for 24 h. Blank nanoparticles were taken as control. The next day, the wells were washed twice with sterile PBS for 5 min. The cells were fixed with 4% paraformaldehyde solution for 30 min. Later, the cells were permeabilized with 0.25% Triton-X for 15 min and washed with PBS two times for 5 min. The blocking was carried out with 2% albumin in PBS for 30 min. After binding, the cells were incubated with primary antibody 50 µL/mL overnight at 4°C. The next day, cells were washed with PBS two times for 5 min. Later, cells were incubated with anti-rabbit secondary antibody for 1 h at room temperature. After incubation with secondary antibody, they were stained with DAPI for 15 min. The cover clip was then transferred to a glass slide, and nail polish was applied to the edges of the coverslip to prevent it from drying

### 2.17. Quantification of p65 and HIF-1α

CaCo-2 and HT-29 cells (pre-treated for 12 h with 10% oxygen) were treated with free drugs, PLGA nanoparticles, and albumin-loaded nanoparticles for 6 h. Whole-cell lysates were prepared and assayed for HIF-1α and p65 levels using an Invitrogen HIF1a Human ELISA Kit (EHIF1A) and an NF-κβ p65 (Total) Human ELISA Kit (KHO0371), used according to the manufacturer’s instructions at 450 nm, using a BioTek optical plate reader. Optical density was converted to concentration (pg mL-1247) using the standard calibration curve provided in the manufacturer’s protocol.

### 2.18. In Vivo Azoxymethane (AOM)/Dextran Sodium Sulfate (DSS) Model of Colon Cancer

The animal experimental protocols were approved by the institutional animal ethical committee of the University of Ulster, under Home licence number PL 2768. The experiments were carried in C57 BL/6 mice aged 10–12 weeks. Mice were maintained under controlled conditions such as optimum room temperature, humidity, and light (12/12 h light/dark cycle), and permitted ad libitum access to standard mouse chow and tap water. The mice were kept in these conditions up to 7 days prior to experiments.

### 2.19. Carcinogenesis Induction

Animals were divided into four groups (*n* = 6 per group): Control (positive control), Blank, PIC, and PIC–BSA NP groups, respectively. All animals were administrated with a single intraperitoneal injection of AOM (7.5 mg/kg) in the beginning. One week after the AOM injection (set as Day1), the animals began to receive 2.5% w/v DSS in the drinking water for 8 consecutive days. We calculated that the non -toxic daily dose of PIC was approximately 40 mg/kg, and a similar dose was used for both PIC and PIC–BSA NPs. The weight of each mouse was recorded on a daily basis, and colon tumor size and number were recorded post-mortem [58,59].

### 2.20. Statistical Analysis

Results were expressed as mean ± standard error of the mean (SEM) for a series of experiments. Data were assumed to be normally distributed and statistical analyses were carried out using Prizm Graph Pad V6 software (Graph Pad, San Diego, CA, USA). A paired *t*-test was used for comparisons of paired treatments between two groups, unpaired *t*-tests for comparisons of unpaired treatments between two groups, and one-way ANOVA using Bonferroni multiple comparisons tests for treatments of three groups or more. *p*-values ≤ 0.05 were considered to be significant.

## 3. Results

The effects of ethanol concentration, albumin concentration, glutaraldehyde, and amount of drug on particle size, polydispersity, and zeta potential were studied. BSA between 50 to 200 mg was dissolved in 2 mL of purified distilled water. Subsquently, PIC (10, 20, and 30 mg) was dissolved in 8 mL ethanol, which was added dropwise into the aqueous albumin solution under magenetic stirring (500 rpm), resulted in formation of opalescent suspension spanotanously at room temperature. After this desolvation process, 0.11 mL of 8% glutaraldehyde in water (*v*/*v*) was added to cross the dessolved PIC–BSA NPs. The calibration curve of PIC was plotted, as shown in Figure S1, in methanol (1:9) and absorbance was measured at 325 nm.

### 3.1. Effects of Ethanol, Albumin, and Glutaraldehyde Concentrations on Particle Size, Polydispersity Index and Zeta Potential

In order to influence the resulting particle size, the rate of the ethanol addition during the desolvation procedure, as well as the pH value of protein solution used for desolvation, was varied—see Figures S1 and S2. As previously observed [48], in the set of experiments performed, the rate of ethanol addition did not show significant influence on the average diameter of the resulting nanoparticle or polydispersity index. Similarly, the current study showed that PIC-loaded albumin NP was fabricated with 4, 8, and 12 mL of ethanol. The particle size was found to be in the range 227–238 nm and the polydispersity index was in the range of 0.154–0.172. Though, zeta potential showed a slight decrease in value from −13 to −18 mV.

PIC-loaded albumin NP was formulated with various concentrations of albumin to observe its effect on parameters such as particle size, polydispersity, and zeta potential. A significant (*p* < 0.01) increase in particle size from 211 to 252 nm was observed when albumin content increased from 0.5 to 2%. The particle size further increased to 294 nm as the concentration of albumin increased to 3.5%. Similarly, the polydispersity index increased (from 0.11 to 0.27) and the zeta potential (from −19 to −12 mV), along with the increase in albumin ratio. Both observations are in good agreement with earlier work [48], which suggests that a reduction in the size of the nanoparticles could be due to an increase in ionization of BSA.

The glutaraldehyde cross-linking procedure was identified as a crucial parameter for evaluation of biodegradability and drug release of the nanoparticles. An increase in glutaraldehyde concentration resulted in increased particle size, but decreased the polydispersity index, as well as zeta potential. Particle size increased from 225 to 257 nm (*p* < 0.05), and from 257 to 391 nm (*p* < 0.001). This observation is in good agreement with earlier work [60] which suggests that a stable formulation of the nanoparticle can be obtained at pH 8 and 8% glutaraldehyde concentration. The polydispersity index decreased from 0.20 to 0.14 (*p* < 0.001), and the zeta potential reduced from −13 to −18 mV (*p* < 0.01). The latter is in good agreement with the earlier work by Li et al. [48].

### 3.2. The Effect of Drug Content

The effect of addition of PIC to BSA was followed through the corresponding increase in size of the nanoparticle, and increased zeta potential was observed (Figure S3A,C). The particle size increased from 210 to 257 nm (*p* < 0.001) as the amount of drug increased from 10 to 20 mg. Similarly, particle size increased to 288 nm when drug content increased to 30 mg (*p* < 0.05). The polydispersity index increased from 0.11 to 0.18 (*p* < 0.01) as drug amount increased (Figure S3B). Likewise, zeta potential increased from –20 to 8 mV (*p* < 0.001) with an increase in PIC amount in the formulation. A valid interpretation for this has been previously suggested [61,62]. An increase in the amount of drug leads to an increase in viscosity of the organic phase, and decreased dispersion of the organic phase into the aqueous phase, resulting in the larger size of nanoparticles.

### 3.3. Drug Entrapment Efficiency and Percent Yield

In previous studies, a higher percentage of drug entrapment was obtained for coated nanoparticles as compared to plain nanoparticles, which could be due to minimum repulsion between drug and polymer [63]. Similarly, in the current study, the pattern of entrapment efficiency of PIC-loaded albumin NP showed a significant (*p* < 0.01) increase in entrapment efficiency as the concentration of albumin increased (Figure S3D). An increase in entrapment efficiency (approximately 65%) was seen as the albumin content increased from 0.5 to 2% (*p* < 0.01). However, the entrapment efficiency was found to be 64% when albumin concentration increased to 3.5% (*p* < 0.01). A reduction in entrapment efficiency from 60 to 39% occurred as the content of drug increased to 30 mg (approximately 39%) (Figure S3E). This implies that an increase in drug amount decreases the entrapment efficiency, because higher amounts of drug would require more amounts of albumin to encapsulate [64].

The effects of albumin concentration and drug content were evaluated on the percentage yield. Figure S3F shows an increase in percentage yield upon increased albumin content in the formulation. Percentage yield increased from 50 to 64% when albumin concentration increased from 0.5 to 2% (*p* < 0.05). However, there was a slight increase in yield as the albumin concentration increased in the formulation to 3.5%. This increase in percentage yield may be due to the availability of a higher amount of albumin for the same amount of PIC. A similar trend for percentage yield can be seen in Figure S3G, where percentage yield increased from 42 to 67% when the drug amount increased (*p* < 0.05). The yield slightly increased to 68% when the amount of drug increased to 30 mg. This observation is in good agreement with earlier work [65], which suggested that the variation in drug/polymer ratios might have an influence on the physical characteristics of the microspheres, and the increasing amount of the polymer might result in decreased drug desolvation.

### 3.4. In Vitro Drug Release and Solubility of PIC-Loaded Albumin NPs

PIC nanoparticles were formulated with 0.5, 2, and 3.5% concentrations of albumin. These batches show burst release of PIC from nanoparticles after 24 h (Figure S4A). It was noted that an increased amount of albumin decreased the release of PIC from nanoparticles. The release of 34, 29, and 22% was observed after 24 h in these batches. Moreover, 70, 62, and 55% release of PIC was reported after 168 h. Larger particles show a decrease in release, presumably due to the higher albumin content. Increase in albumin concentration contributes to a decrease in the in vitro release of the drug [63].

In further experiments, the effect of the increased amount of PIC from 10 to 30 mg on release profile was evaluated (Figure S4B). The result showed a burst release of PIC from nanoparticles. After 24 h, 50, 37, and 32% release of PIC was reported. After 168 h, the PIC release was 77, 63, and 50% in batches formulated with 10, 20, and 30 mg of PIC, respectively. A similar trend of release was previously reported by Azizi et al. [64]. Rapid dissolution of PIC present on the surface of albumin may cause the burst release in the dissolution media.

Aqueous solubility is one of the most important physicochemical factors for drug absorption. Furthermore, good aqueous solubility has been reported to play a major role in enhancing the pharmacokinetic properties of drugs, including their bioavailability, membrane flux, and therapeutic efficacy [66]. In the present study, the water solubility of PIC in both free and encapsulated forms was determined by UV-spectroscopy at 325 nm. The vials (Figure S5A) showed an enhanced solubility of PIC when formulated as PIC-loaded albumin NP vs. free PIC in water (Figure S5B). It is evident that tube A produced a uniform dispersion of PIC-loaded albumin NP (Figure S5A), whereas free PIC was sparingly soluble (Figure S5B). Tube B (Figure S5) showed turbidity of particles, whereas tube A showed a clear solution. The solubility of free PIC was 17.87 µg/mL, whereas that of PIC-loaded albumin NP was 29.26 µg/mL, which represents an approximate double increase in aqueous solubility of PIC-loaded albumin NPs as compared to free PIC, as shown in the absorbance spectra of PIC-loaded albumin NPs and free PIC (Figure S5B).

### 3.5. Morphology and Cellular Uptake of PIC-Loaded Albumin NPs

The size of the nanocarrier system plays a crucial role in drug delivery, as nanoparticles up to 400 nm preferentially affect the tumor microenvironment via the enhanced permeability and retention (EPR) effect [67]. Morphological evaluation of free PIC and PIC-loaded albumin NPs was assessed by scanning electron microscopy (SEM). Figure S4C shows the SEM images of free PIC and different batches of nanoparticles such as F1 (PIC 10 mg), F2 (PIC 20 mg), and F3 (PIC 30 mg). Free PIC represents the random and solid structure of the drug, whereas images of the nanoparticles show spherical and smooth surfaces. Image F1 demonstrates monodisperse and smooth textured appearance. However, images F2 and F3 show larger particle sizes and partially disperse textures. Therefore, from the above SEM images, it can be concluded that an increase in the drug content increases the particle size of nanoparticles. Thus, to achieve intracellular targeting, a smaller particle size ranging from 0.1 µm to 100 nm is desirable.

**Figure 1:**
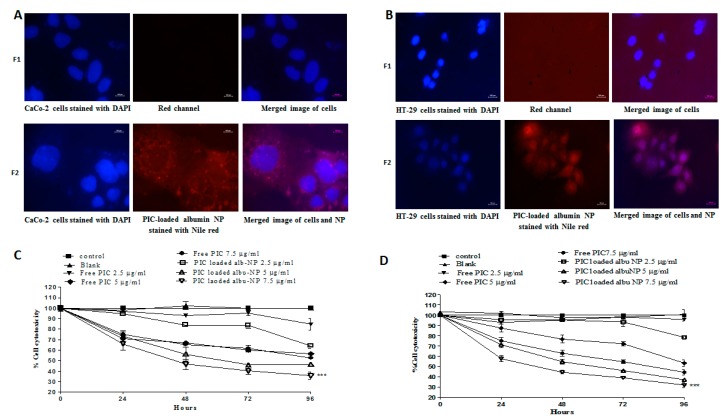
Cellular uptake and in vitro cytotoxicity of PIC-loaded nanoparticle. Cellular uptake of optimized PIC-loaded albumin nanoparticles (NPs) in CaCo-2 and HT-29 cells at 100×. Cellular localization of Nile red-coated PIC-loaded albumin NPs was observed in CaCo-2 (**A**) and HT-29 (**B**) cells and visualized by overlapping under fluorescent microscopy. The red fluorescent can be observed in the microscopic images F2 in Figure 6A,B. The cytotoxicity assay was evaluated on CaCo-2 (**C**) and HT-29 (**D**) cell lines; 40 × 10^4^ cells were seeded on 24-well plates and treated with 2.5, 5, and 7.5 µg/mL of PIC-loaded albumin NPs and an equivalent amount of free PIC. The absorbance was calculated after 24, 48, 72, and 96 h. PIC-loaded albumin NPs show higher cell cytotoxicity as compared to free PIC. Values are mean ± standard error of the mean (SEM) with *n* = 3. *** *p* < 0.001 compared with same amount of free drug.

Furthermore, the cellular uptake of optimized PIC–BSA NPs in colon cancer cell lines (CaCo-2 and HT29) was evaluated by using phase-contrast microscopy. Image F1 (Figure 1A,B) shows CaCo-2 and HT-29 cells labeled by DAPI. Image F2 (Figure 1A,B) shows CaCo-2 and HT-29 cells labeled with Nile red-coated PIC-loaded albumin NPs. The merged images in F1 show nuclei stained by DAPI, whereas merged images in F2 show intense Nile red-labeled fluorescent nanoparticles surrounding the nucleus, indicating that a sufficient amount of the PIC–BSA NPs was efficiently internalized into CaCo-2 and HT29 cells.

Overall, NPs are efficiently taken up by cancer cells via endocytosis, owing to the only nano-size effect. Furthermore, the 60 kDa glycoprotein (gp60) is highly expressed in endothelial cells around tumors, and albumin carriers efficiently bind to gp60, forming caveolae of albumin–gp60 complex. This complex traverses to the tumor interstitium via the so-called gp60-mediated active transcytosis pathway [32]. It is expected that our PIC–BSA NPs will have great potential to accumulate in tumor sites when applied intravenously

### 3.6. NMR Spectrum of PIC-Loaded Albumin NPs

The ^1^ H NMR study (in DMSO-d6) of PIC in albumin nanoparticles revealed that the signal due to four hydroxyl groups appeared as a broad spectrum (brs) around 9.1 ppm, whereas the two trans olefinic protons a and b resonated at around 6.87 and 6.75 ppm, respectively. The four singlet aromatic protons (d, f, g, h) appeared at around 6.96, 6.71, and 6.11, respectively. These data confirm the PIC in the albumin nanoparticle structure, as shown in Figure S5C. Like many other drug molecules [61], PIC interacts with a tryptophan residue (Trp 212) located inside the hydrophobic pocket of the BSA.

### 3.7. In Vitro Cytotoxicity and Cell Migration of PIC–BSA NPs

The cytotoxicities of PIC–BSA NPs and free PIC in colorectal cancer cell lines (CaCo-2 and HT29 cells) were assessed using the MTT assay. The application of PIC–BSA NPs significantly (*p* < 0.001) increased cell cytotoxicity as compared to the cytotoxicity afforded by free PIC (Figure 1C,D). Similarly, time- and dose-dependent responses were observed in PIC–BSA NPs and free PIC cytotoxicity at 24–96 h incubation. This observation is in accordance with previous reports [32,68], which suggest that PIC-loaded albumin NPs sustained the cytotoxic action of the drug due to the enhanced penetration and retention effect inside the colorectal cells. It also enhanced the solubility, which in turn enhances the half-life and bioavailability [68] due to a reduction in particle size of the PIC. Therefore, based on the above results, it can be concluded that PIC-loaded albumin NPs improve the cytotoxicity potential due to a reduction in particle size, which suggests that albumin nanoparticulate formulation enhances the biological therapeutic efficacy of PIC over free PIC.

**Figure 2:**
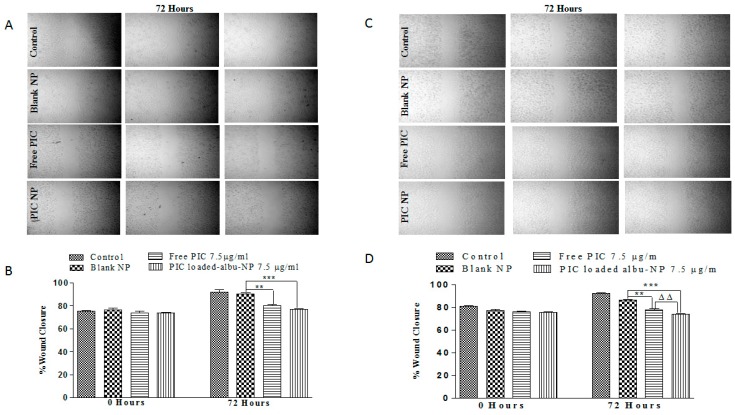
Effect of PIC-loaded albumin nanoparticles on migration potential. Effect of PIC-loaded albumin NPs and free PIC on migration potential of CaCo-2 cell lines. The above image in (**A**) demonstrates the effect of free PIC and PIC-loaded albumin NPs on wound closure after 72 h of treatment. PIC-loaded albumin NPs demonstrate more void space than free PIC in the wound scratch of cell lines as compared to control and blank NPs. (**B**) depicts a significant variation of anti-migration activity of free PIC (*p* < 0.01) and PIC-loaded albumin NPs (*p* < 0.001) after 72 h of treatment at equal dose. Values are mean ± SEM with *n* = 3. ** *p* < 0.01, *** *p* < 0.001 compared with blank NPs. Similarly, (**C**,**D**) represents the effect of PIC-loaded albumin NPs and free PIC on migration potential of HT-29 cell lines. (**C**) shows the effect of free PIC and PIC-loaded albumin NPs on wound closure after 72 h of treatment. PIC-loaded albumin NPs demonstrate more void space than free PIC in the wound scratch of cell lines as compared to control and blank NPs. (**D**) shows a significant variation of anti-migration activity of free PIC (*p* < 0.01) and PIC-loaded albumin NPs (*p* < 0.001) after 72 h of treatment at equal dose. Values are mean ± SEM with *n* = 3. ** *p* < 0.01, *** *p* < 0.001 compared with blank NPs and ^ΔΔ^ *p* < 0.01 compared with the same dose of free PIC.

To investigate the reduction in cell migration, a wound-healing assay was performed to observe the effect of PIC–BSA NPs on cell migration. The level of wound healing was measured by an average decrease in the distance between the edges of the wounds at different time points. The wound-healing assay revealed that CaCo-2 and HT29 cells that were not treated with PIC–BSA NPs effectively healed the wound, with a reduction to 80% and 78% of the original distance in CaCo-2 and HT29 cells after 72 h. However, treatment with 7.5 ug/mL PIC–BSA NPs significantly (*p* < 0.001) repressed wound healing in both CaCo-2 and HT29 cells (Figure 2A–D) as compared to free PIC. Thus, PIC-loaded albumin NPs show strong inhibition of migration of the tumor cells in both CaCo-2 and HT29 cells, but free PIC has less efficiency against migration. Similar observations were previously reported [69], which indicate that like any other tumor cells, colon cancer cells also migrate from one side to another. Therefore, albumin nanoparticles have high efficiency against the migration of cancer cells to prevent the metastasis of colon cancer cells.

### 3.8. Colony-Forming and Invasion Assay

**Figure 3:**
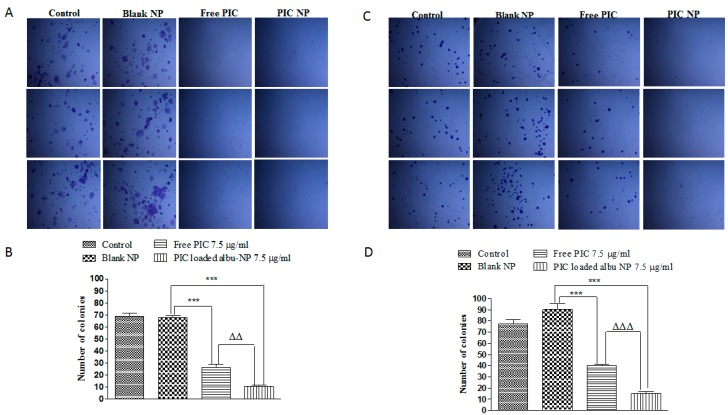
Effect of PIC-loaded albumin nanoparticles on colony formation effect of PIC-loaded albumin NPs and free PIC on colony formation ability of CaCo-2 cell lines. The above images in (**A**) show a pattern of colon formation in control, blank NPs, free PIC, and PIC-loaded albumin NPs treated the group as demonstrated in the microscopic images. (**B**) represents significant (*p* < 0.01) variation in colony formation upon treatment with free PIC and PIC-loaded albumin NPs. The PIC-loaded albumin NPs show a significantly lower number of colonies than free PIC. Values are mean ± SEM with *n* = 3. *** *p* < 0.001 compared with blank NPs. ^ΔΔ^ *p* < 0.01 compared with the same dose of free PIC. Similarly, (**C**,**D**) represents the effect of PIC-loaded albumin NPs and free PIC on colony formation ability of HT-29 cell lines. (**C**) shows a pattern of colony formation in control, blank NPs, free PIC, and PIC-loaded albumin NPs treated the group as demonstrated in the microscopic images. (**D**) represents (*p* < 0.001) variation in colony formation upon treatment with free PIC and PIC-loaded albumin NPs. The above figures reveal that PIC-loaded albumin NPs show a significantly lower number of colonies than free PIC. Values are mean ± SEM with *n* = 3. *** *p* < 0.001 compared with blank NPs. ^ΔΔΔ^ *p* < 0.001 compared with the same dose of free PIC.

Cancerous cells are prone to the formation of colonies from a single cell due to their inherent property of uncontrolled division and proliferation [70]. In the present study, clonogenic assay was performed using 7.5 µg/mL of PIC-loaded albumin NPs and free PIC. A significant (*p* < 0.01) decrease in the colonies was observed for both free PIC and PIC–BSA NPs in CaCo-2 and HT29 cells. Interestingly the number of colonies was found to be less than 50 in numbers for the above groups. Hence, the nanoparticle formulation showed more significant (*p* < 0.01) inhibition of colony formation as compared to free PIC (Figure 3A–D).

**Figure 4:**
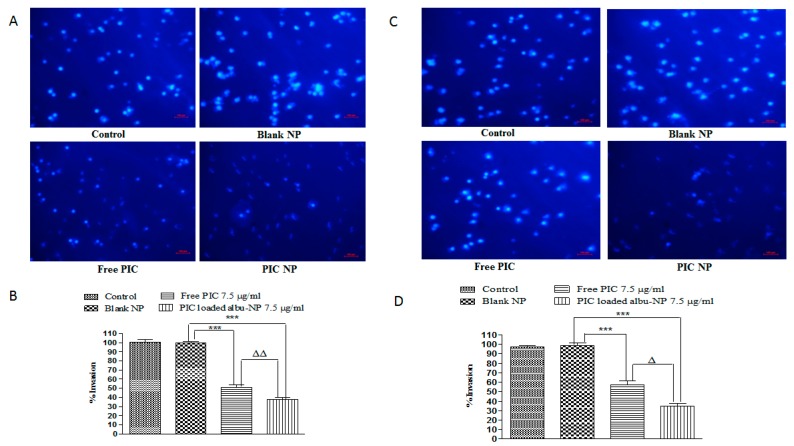
Effect of PIC-loaded albumin nanoparticles on invasion potential Effect of PIC-loaded albumin NP and free PIC on invasion potential of CaCo-2 cell lines. (**A**) represents anti-invasive activity of free PIC and PIC-loaded albumin NPs after treatment. The cells which appear bright are the ones which invaded through the membrane. PIC-loaded albumin NPs show a significantly (*p* < 0.01) lower percentage of invasion than free PIC. (**B**) shows that PIC nanoparticles represent significantly less invasion than free PIC (*p* < 0.01). Values are mean ± SEM with *n* = 3. *** *p* < 0.001 compared with blank NPs. ^ΔΔ^ *p* < 0.01 compared with the same dose of free PIC. Similarly, (**C**,**D**) represents anti-invasive activity of free PIC and PIC-loaded albumin NPs after treatment in HT-29 cells. The cells which appear bright are the one swhich invaded through the membrane. (**C**) shows PIC-loaded albumin NPs show a significantly (*p* < 0.05) lower percentage of invasion than free PIC. (**B**) shows that the PIC nanoparticles have significantly less invasion than free PIC (*p* < 0.05). Values are mean ± SEM with *n* = 3. *** *p* < 0.001 compared with blank NPs. ^Δ^ *p* < 0.05 compared with the same dose of free PIC.

Furthermore, the effect of PIC-loaded albumin NPs on the invasion activity of colorectal cancer cell lines (CaCo-2 and HT-29 cells) was assessed by using invasion assay. Both CaCo-2 and HT29 cells were treated with 7.5 µg/mL of PIC–BSA NPs and the same dose of free PIC. The efficiency of PIC-loaded albumin NPs in inhibiting invasion was significantly (*p* < 0.01) greater than that afforded by free PIC (Figure 4A,C). The inhibition of invasion was significantly (*p* < 0.001) lower (38%) in CaCo-2 cells treated with PIC–BSA NPs as compared to free PIC (51%) (Figure 4B). Additionally, PIC–BSA NPs showed increased efficiency against invasion as compared to free PIC in HT-29 (Figure 4D). Cell invasion inhibition was significantly less (*p* < 0.05) in the PIC-loaded albumin NP group (34%) than that treated with free PIC (57%) in HT-29 cells. The results indicate that PIC-loaded albumin NPs are more effective against the invasion of colon cancer cells (CaCo-2, and HT29 cells) due to their enhanced and targeted activity.

### 3.9. Immunostaining of p65 and HIF-1α

**Figure 5:**
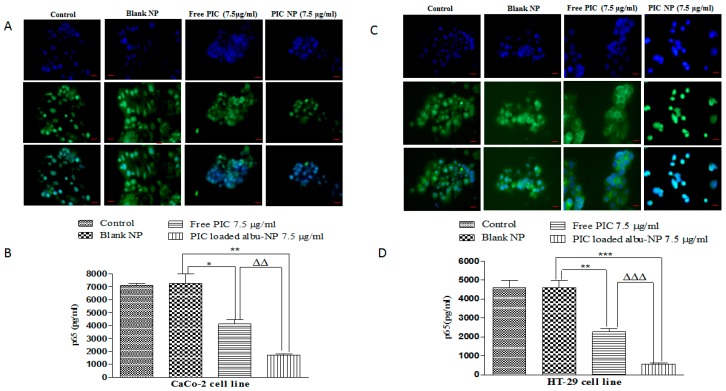
Effect of PIC-loaded albumin nanoparticles on the expression of p65. Effect of PIC-loaded albumin NPs and free PIC on the expression of p65 in CaCo-2 at 40×. (**A**) were captured under bright field microscopy. Control and blank images show induction of p65, whereas images under free PIC and PIC-loaded albumin NPs show a reduced level of p65. (**B**) shows a significant (*p* < 0.01) reduction in the level of p65. Values are mean ± SEM (*n* = 3). * *p* < 0.05 and ** *p* < 0.01 as compared to control. ^ΔΔ^ *p* < 0.01 compared to same amount of free PIC. Similarly (**C**) shows images captured under bright field microscopy in HT-29 cells at 40X. Control and blank images show induction of p65 whereas images under the free PIC and PIC-loaded albumin NP show a reduced level of p65. (**D**) reveals significant reduction in the level of p65. Values are mean ± SEM (*n* = 3). ** *p* < 0.01 and *** *p* < 0.001 as compare to control. ^ΔΔΔ^ *p* < 0.001 compared to same amount of free PIC.

Overexpression of pro-inflammatory cytokines is responsible for the induction of transcription factor nuclear factor kappa beta (NF-kβ) and hypoxia-inducible factor-1α (HIF-1α) [9,10]. Furthermore, overactivation of NF-κβ and HIF-1α leads to proliferation, migration, and invasion at the tumor site [11,12,13]. It is well documented that pro-inflammatory cytokines induce aberrant activation of NF-κβ and HIF-1α in ulcerative colitis and colorectal cancer patients [14,15,16,17]. In the present study, the effect of PIC–BSA NPs and free PIC on the expression of p65 was studied by staining both CaCo-2 and HT29 cells with p65 antibody and viewed under bright field microscopy. A significant (*p* < 0.01) reduction in the level of p65 expression was observed in the cells when they were treated with 7.5 µg/mL of PIC–BSA–NP as compared to the cells treated with 7.5 µg/mL free PIC in both cell lines (Figure 5A,B). These findings were qunatiated using human ELISA kit for p65 which are in agreement with the immunostaining and shows significant reduction in levels of p65 in both CaCo-2 and HT29 cells (Figure 5C,D).

**Figure 6:**
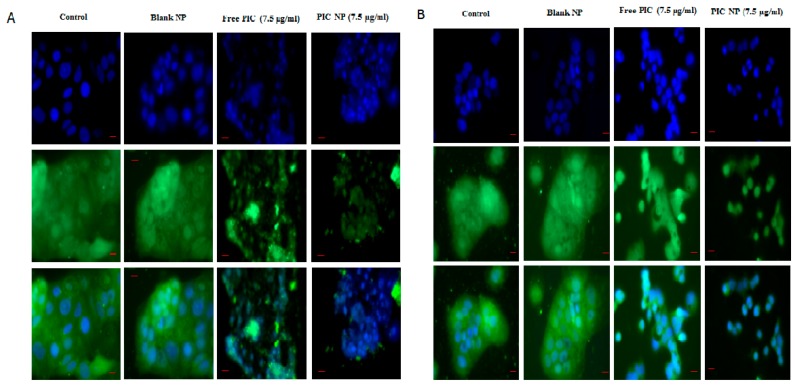
Effect of PIC-loaded albumin nanoparticles on the expression of HIF-1α. Effect of PIC-loaded albumin NPs and free PIC on the expression HIF-1α in CaCo-2 (**A**) and HT-29 (**B**) at 40×. The above images shows induction of HIF-1α in control and blank transfected group, whereas images under free PIC and PIC-loaded albumin NPs show a reduced level of HIF-1α.

Furthermore, the effect of PIC–BSA NPs and free PIC on the expression of HIF-1α was investigated by staining both CaCo-2 and HT29 cells with HIF-1α antibody and viewed under bright field microscopy. Reduced expression of HIF-1α was found in cells treated with PIC–BSA NPs as compared to free PIC (Figure 6A,B). An overstimulated expression of HIF-1α was found in control and cells treated with blank NPs (Figure 6A,B). The results indicate that PIC–BSA NPs are effective against the downregulation of HIF-1α that leads to the reduction in proliferation, migration, and invasion of the tumor.

### 3.10. Inhibition of Experimental Colitis

**Figure 7:**
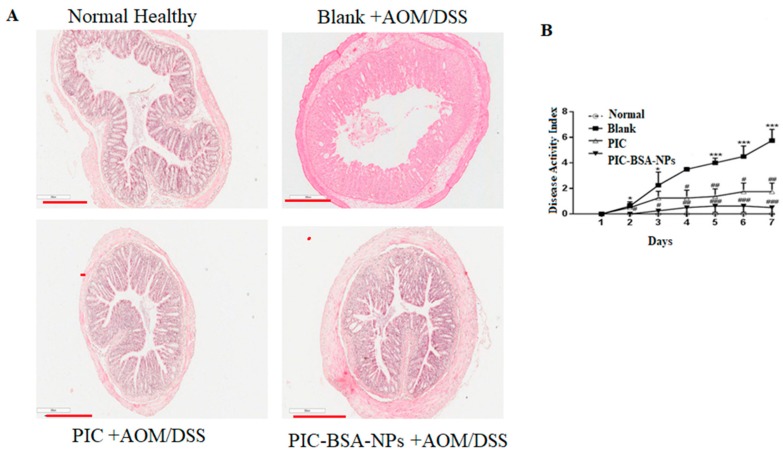
Effect of PIC-loaded albumin nanoparticles on inhibition of experimental colitis. Effect of PIC-loaded albumin NPs and free PIC on azoxymethane/dextran sodium sulphate-induced change in acute colitis (*n* = 10 per group). (**A**) Representative hematoxylin and eosin-stained histological sections of control (or negative control), healthy, free PIC, and PIC–BSA NP groups. (**B**) PI–BSA NPs prevent colitis, expressed as disease activity index. Data from the control (or negative control) group are all zeros from day 1 to day 14.

After the administration of DSS, the animals showed a remarkable increase in disease manifestation, such as apparent diarrhoea and rectal bleeding, commencing from day 4 in the blank group (Figure 7A). As treatment continued, the presence and development of inflammation were more severe. During the acute phase (7 days), H&E staining of colon tissue from the blank group showed an increase in severity, such as inflammatory lesion throughout the colon, complete loss of crypts, surface erosion with inflammatory exudates, inflammatory infiltrate, and submucosa oedema. In contrast, in the free PIC and PIC–BSA NP groups, animals had tightly packed glands with a normal amount of goblet cells (Figure 7A). The disease severity, scored as DIA, shows its highest level on day 7. Figure 7B exhibits a significant effect of PIC–BSA NPs on the reduction of DIA score (*p* < 0.05). This inhibition of experimental colitis by PIC-fabricated nanoparticles was not only apparent during DSS treatment, but also very evident after cessation of DSS administration (i.e., day 7). These results suggest that PIC in nano-formulation significantly reverses colitis.

### 3.11. Protection from Colitis-Associated Colon Carcinogenesis

**Figure 8:**
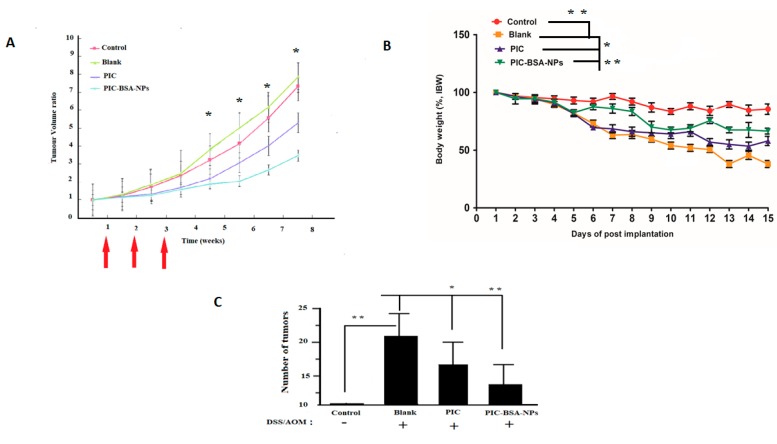
Effect of PIC-loaded albumin nanoparticles on tumor size, number of tumors, and body weight. The effect of PIC-loaded albumin NPs and free PIC on azoxymethane/dextran sodium sulphate-induced colon carcinogenesis (*n* = 10 per group). (**A**) The size of the tumor reduced significantly (*p* < 0.05) in the PIC–BSA NP group compared to the control (negative or blank). (**B**) Percentage body weight reduction was significant (*p* < 0.05) in PIC–BSA NPs. (**C**) Number of tumors reduced very significantly (*p* < 0.05) in PIC–BSA NPs compared to control, blank, and free PIC, respectively.

In colorectal cancer chemoprevention animal studies, AOM (mutagenic agent/and/or DSS (a proinflammatory agent)) is often used as an experimental model [71,72] In this study, we used the AOM/DSS model to elucidate the efficacy of free PIC and its fabricated form into nanoparticles for effective anti-colorectal cancer action. Figure 8C shows colon carcinogenesis data. The PIC–BSA NP group showed a highly significant reduction in the number of colon tumors and the size of tumors as compared to control, blank, and PIC groups, respectively. Similarly, Figure 8B shows body weight percentage changes in different experimental groups. Compared with control, which had a slow weight gain, the blank group had significant weight reduction. This reduced weight remained for over 14 days after cessation of DSS. Both the PIC and PIC–BSA NP groups significantly increased percentage weight gain compared with the blank group, respectively. However, weight gain by the PIC–BSA NP group was highly significant as compared to the control and PIC groups.

### 3.12. Long-Term Follow-Up Study

Furthermore, tumor size increased with time in all four treatment groups (Figure 8A). The tumors gradually increased from the beginning in the blank group, whereas, the tumor in the other groups was relatively suppressed during the first three weeks of the treatment period. Among all groups, the PIC–BSA NP group showed the most delayed tumor growth, with the initiation of rapid tumor growth in the fourth week in the other groups. In addition, tumors in the PIC–BSA NP group showed the slowest growth rate among all. Starting in the fifth week, the tumor–volume ratio in the PIC–BSA NP group was significantly (*p* < 0.05) lower than those in the control, blank, and PIC groups, respectively. Similarly, PIC–BSA NPs were highly significant when compared with free PIC in tumor–volume ratio. Previous studies were consistent with our findings [73]. These results suggest that PIC fabricated into nanoparticles could improve and enhance the anticancer action of PIC by overcoming its absorption limitations.

## 4. Discussion and Conclusions

Piceatannol is a naturally occurring polyphenolic stilbene found in various fruits and vegetables, and has been reported to exhibit anticancer and anti-inflammatory properties. The present study aimed to design and characterize BSA NPs as a potential carrier for drug delivery of piceatanol. A major obstacle in the clinical use of piceatannol (PIC) is its reduced bioavailability because of the drug’s low solubility (0.097 mg/mL) and low internal absorption. Here, we report for the first time the encapsulation of PIC in a biodegradable non-toxic carrier (albumin) as PIC–BSA NPs, and utilize a murine model of chemically-induced colon cancer to demonstrate that the nanoparticle formulation is better at lowering the number of colon tumors and size of the tumors as compared to PIC alone. Furthermore, we observed a significant reduction in inflammation induced by chemical colitis in mice by PIC–BSA NPs.

The designed PIC–BSA NPs were prepared by the desolvation technique. The developed formulation was characterized by assessing its morphology, zeta potential, encapsulation efficiency, and controlled drug release profile. BSA is a natural protein able to form complexes with many drugs. It is biodegradable, non-toxic, and nonimmunogenic, making it a good drug delivery system [36]. The PIC–BSA NPs were obtained with the continuous dropwise addition of ethanol to BSA solution, due to the diminishing water solution of BSA. The resultant nanoparticles were not sufficiently stable, since desolvation with ethanol is a reversible process. Glutaraldehyde was used to harden the coacervates by cross-linking. In the cross-linking, amino groups of lysine and guanidino groups in arginines enter into a condensation reaction with the aldehyde group of glutaraldehyde. This process does not appear to have a significant effect in particle size [74].

Our present pinoering research reports, for the first time, the encapsulation of PIC in a biodegradable non-toxic carrier (albumin) as PIC–BSA NPs. Our observations indicate that the particle size, morphology, and stability analysis of the PIC–BSA NPs are quite stable, spherical in shape, and exsist as a monodisperse system. These findings support that the drug incorporation did not alter the the synthesized nanoparticles in terms of size and shape. Our PIC–BSA NPs were within the optimal nanoparticle size range for drug delivery applications. The cellular uptake and drug release studies, together, demonstrated that the time required for the total release of the drug allows nanoparticles to penetrate into the cells, where drug release would be promoted by BSA degradation by intracellular proteases [74] and lysosomal conditions. The PIC deposition effect is expected to be more pronounced in, or near, hypoxic conditions of lower pH, since acidity conditions near the isoelectric point of BSA (pH 5.4) lead to a sharp decline in its solubility. Our results confirm the nontoxic nature of the BSA nanocarriers, and further indicate improved anti-colon cancer efficacies of PIC–BSA NPs as compared to free PIC via in vitro cytotoxicity assay, migration studies, and colony-forming and invasion assays.

Furthermore, results from the MTT assay indicate the nontoxic nature of the BSA nanocarriers; our findings indicate that BSA encapsulation sufficiently enhanced the anticancer efficacy of PIC in cancer cells as compared to free PIC.

Additionally, the PIC–BSA NPs showed significantly enhanced downregulation with transcription factor p65, which is a protein encoded in humans by the *RELA* gene, which is known to be positively associated with almost all types of cancers. We also showed, for the first time, that PIC can downregulate the expression of hypoxia-associated transcription factor HIF-1 alpha, which plays a major role in cancer biology, especially in the areas of angiogenesis, cell survival, and tumor invisaion. PIC, when encapsulated as albumin nanoparticles, resulted in a higher degree of suppression of both p65 and HIF-1 alpha as compared with free PIC. This may suggest that the PIC–BSA NPs are useful as vehicles for the intracellular release of PIC.

Our in vivo studies in a murine model of chemically-induced colon cancer indicate that PIC–BSA NPs have a better theraputic outcome as compared to free PIC in terms of lowering the number of colon tumors and size of the tumors. This could be attributed to its enhanced ability to downregulate p65 and HIF-1 alpha.

## 5. Future Work

The present study proposes that the anti-colon cancer potential of PIC could be improved by loading it to a protein-based carrier for future clinical applications.

Our future studies will involve comparing the therapeutic potential of PIC–BSA NPs with clinically used chemotherapeutics, and also investigation of surface modification of PIC–BSA NPs to enhances their inflammation- and cancer-targeting ability.

## Supplementary Materials

The following are available online at https://www.mdpi.com/2072-6694/12/1/113/s1, Figure S1: Calibration curve of Piceatannol, Figure S2: Effect of ethanol, albumin and glutaraldehyde concentrations on particle size, polydispersity index and zeta potential, Figure S3: Effect of increase in drug amount on particle size, polydispersity index, zeta potential, albumin entrapment efficiency and percentage yield., Figure S4: In vitro release and scanning electron microscopy of different batches of nanoparticles, Figure S5: Solubility and NMR spectra of PIC-loaded nanoparticle.
