# Correction: Aljabali, A.A.A.; et al. Albumin Nano-Encapsulation of Piceatannol Enhances Its Anticancer Potential in Colon Cancer via down Regulation of Nuclear p65 and HIF-1α. *Cancers* 2020, *12*, 113

**DOI:** 10.3390/cancers12123587

**Published:** 2020-11-30

**Authors:** Alaa A. A. Aljabali, Hamid A. Bakshi, Faruck L. Hakkim, Yusuf A. Haggag, Khalid M. Al-Batanyeh, Mazhar S. Al Zoubi, Bahaa Al-Trad, Mohamed M. Nasef, Saurabh Satija, Meenu Mehta, Kavita Pabreja, Vijay Mishra, Mohammed Khan, Salem Abobaker, Ibrahim M. Azzouz, Harish Dureja, Ritesh M. Pabari, Ashref Ali K. Dardouri, Prashant Kesharwani, Gaurav Gupta, Shakti Dhar Shukla, Parteek Prasher, Nitin B. Charbe, Poonam Negi, Deepak N. Kapoor, Dinesh Kumar Chellappan, Mateus Webba da Silva, Paul Thompson, Kamal Dua, Paul McCarron, Murtaza M. Tambuwala

**Affiliations:** 1 Department of Pharmaceutical Sciences, Faculty of Pharmacy, Yarmouk University, Irbid 566, Jordan; alaaj@yu.edu.jo; 2 School of Pharmacy and Pharmaceutical Science, Ulster University, Coleraine BT52 1SA, Northern Ireland, UK; Bakshi-H@ulster.ac.uk (H.A.B.); vkahan@gmail.com (M.K.); mm.webba-da-silva@ulster.ac.uk (M.W.d.S.); p.mccarron@ulster.ac.uk (P.M.); 3 Department of Mathematics and Sciences, College of Arts and Applied Sciences, Dhofar University Salalah, Salalah 211, Oman; clonehakkim@gmail.com; 4 Department of Pharmaceutical Technology, Faculty of Pharmacy, University of Tanta, Tanta 31111, Egypt; youssif.hagag@pharm.tanta.edu.eg; 5 Department of Biological Sciences, Faculty of Science, Yarmouk University, Irbid 566, Jordan; albatynehk@yu.edu.jo (K.M.A.-B.); bahaa.tr@yu.edu.jo (B.A.-T.); 6 Department of Basic Medical Sciences, Faculty of Medicine, Yarmouk University, Irbid 566, Jordan; mszoubi@yu.edu.jo; 7 Department of Pharmacy and Biomedical Sciences, School of Applied Sciences, University of Huddersfield, Queensgate, Huddersfield HD1 3DH, UK; mohamed.nasef@hud.ac.uk; 8 School of Pharmaceutical Sciences, Lovely Professional University, Phagwara, Punjab 144411, India; saurabh.21958@lpu.co.in (S.S.); meenu.22252@lpu.co.in (M.M.); kavitapabreja@gmail.com (K.P.); vijaymishra2@gmail.com (V.M.); 9 Department of Gynecology, European Competence Center for Ovarian Cancer, Campus Virchow, Klinikum Charite-Universitatmedizin Berlin, Augustenburger Platz 1, 13353 Berlin, Germany; salem-nuri.abobaker@charite.de; 10 Department of Dermatology, Venerology, and Allergology, Charite-Universitatsmedizin Berlin, Freie Universitat Berlin, Chariteplatz1, 10117 Berlin, Germany; ibrahim.azzouz@charite.de; 11 Department of Pharmaceutical Sciences, Maharshi Dayanand University, Rohtak 124001, India; harishdureja@gmail.com; 12 School of Pharmacy, Royal College of Surgeons in Ireland, D02 YN77 Dublin-09, Ireland; riteshpabari@rcsi.ie; 13 Department of Forensic Science, School of Applied Science, Huddersfield University, Queensgate, Huddersfield HD1 3DH, UK; ashref.dardouri@hud.ac.uk; 14 Department of Pharmaceutics, School of Pharmaceutical Education and Research, Jamia Hamdard, New Delhi 110062, India; prashantdops@gmail.com; 15 School of Pharmacy, Suresh Gyan Vihar University, Jagatpura, Jaipur 302017, India; gauravpharma25@gmail.com; 16 Priority Research Centre for Healthy Lungs, Hunter Medical Research Institute (HMRI), School of Biomedical Sciences and Pharmacy, University of Newcastle, Callaghan, NSW 230, Australia; shakti.shukla@newcastle.edu.au (S.D.S.); Kamal.Dua@uts.edu.au (K.D.); 17 Department of Chemistry, University of Petroleum & Energy Studies, Dehradun 248007, India; parteekchemistry@gmail.com; 18 Departamento de Química Orgánica, Facultad de Química y de Farmacia, Pontificia Universidad Católica de Chile, Av. Vicuña McKenna 4860, 7820436, Macul, Santiago 4860, Chile; nitinbcharbe@gmail.com; 19 School of Pharmaceutical Sciences, Shoolini University of Biotechnology and Management Sciences, Solan 173229, India; poonam.546@shooliniuniversity.com (P.N.); deepaknandkishore.558@shooliniuniversity.com (D.N.K.); 20 Department of Life Sciences, School of Pharmacy, International Medical University, Bukit Jalil, Kuala Lumpur 57000, Malaysia; Dinesh_Kumar@imu.edu.my; 21 School of Biomedical Sciences, University of Ulster, Coleraine BT52 1SA, UK; p.thompson@ulster.ac.uk; 22 Discipline of Pharmacy, Graduate School of Health, University of Technology, Sydney, NSW 2007, Australia; 23 Centre for Inflammation, Centenary Institute, Sydney, NSW 2050, Australia

The authors wish to make the following corrections to this paper [1]: in Figure 2A,C on page 12, some panels appear to show overlapping areas; all images have been now corrected and the different treatment conditions shows the corresponding representative image.

The original version of Figure 2A,C is:


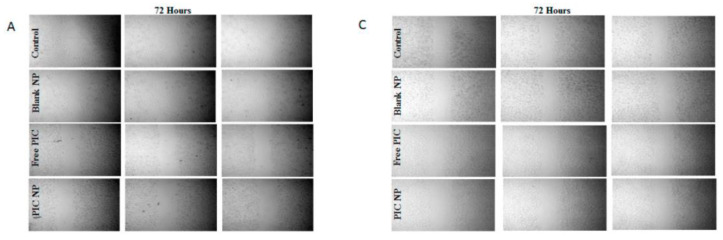


And should be replaced with the following Figure 2A,C:


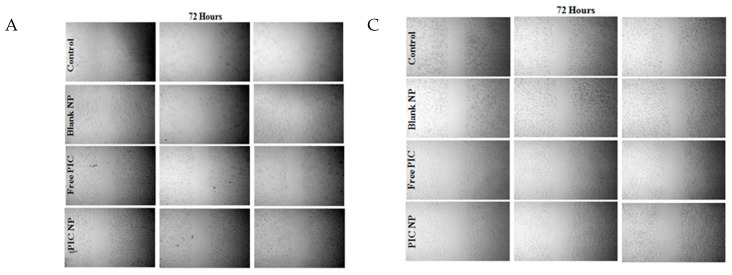


The authors also wish to make the following corrections to this paper [1]: in Figure 4C on page 14, the images for the control and free PIC were taken from the same source due to human error; we have carefully re-assessed all the images and have corrected this error with the appropriate representative images of the control and free PIC groups.

The original version of Figure 4C is:


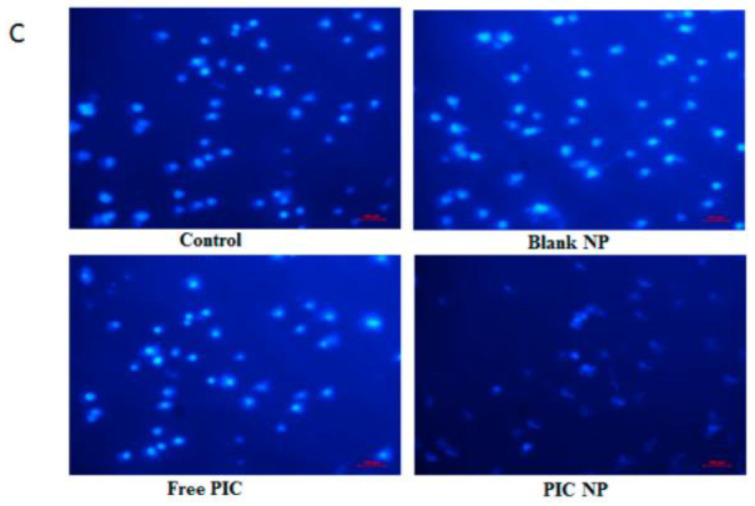


The figure should be replaced with the following Figure 4C with the corrected images:


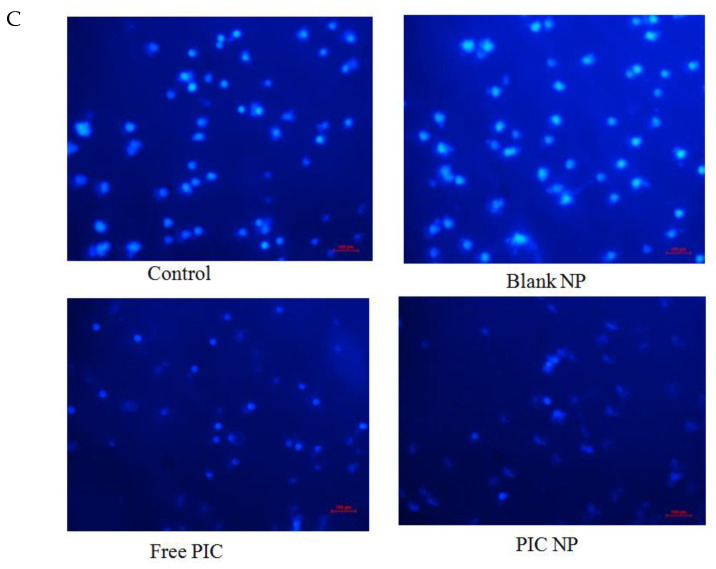


We stress that these errors were purely due to human error and oversight, all corrections done do not change the written proportion of the figure legend, interpretation of the results, or the final conclusion of this manuscript. The manuscript will be updated. The authors would like to apologize for any inconvenience caused. All changes have been reviewed and approved by the Academic Editors.
