# Supplementary material for: Albumin Nano-Encapsulation of Piceatannol Enhances Its Anticancer Potential in Colon Cancer Via Downregulation of Nuclear p65 and HIF-1α
Source: Cancers (Basel). 2020 Jan 1;12(1):113. doi: 10.3390/cancers12010113 (PMC7017258; doi:10.3390/cancers12010113)
Supplement: Supplementary file 1 [file cancers-12-00113-s001.pdf]

Supplementary Materials: Albumin Nano-Encapsulation of Piceatannol Enhances Its Anticancer Potential in Colon Cancer Via Downregulation of Nuclear p65 and HIF-1α

Alaa A. A. Aljabali, Hamid A. Bakshi, Faruk L. Hakkim, Khalid M. Al-Batanyeh, Mazhar S. Al Zoubi, Bahaa Al Trad, Mohamed M Nasef, Saurabh Satija, Meenu Mehta, Kavita Pabreja, Vijay Mishra, Mohammed Khan, Salem Abobaker, Ibrahim M. Azzouz, Harish Dureja, Ritesh M Pabari, Ashref Ali K Dardouri, Prashant Kesharwani, Gaurav Gupta, Shakti Dhar Shukla, Parteek Prasher, Nitin B. Charbe, Poonam Negi, Deepak N Kapoor, Dinesh Kumar Chellappan, Yusuf Haggag, Mateus Webba da Silva, Paul Thompson, Kamal Dua, Paul McCarron and Murtaza M. Tambuwala

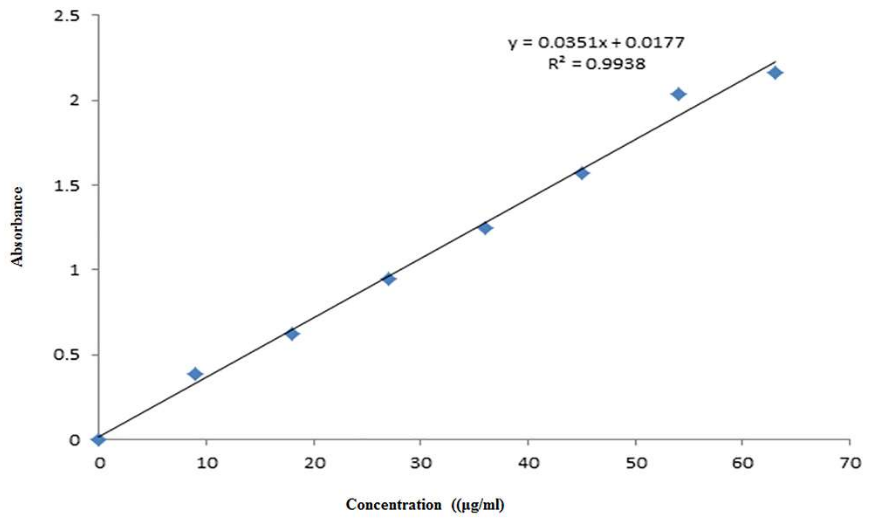

Figure S1. Calibration curve of Piceatannol.

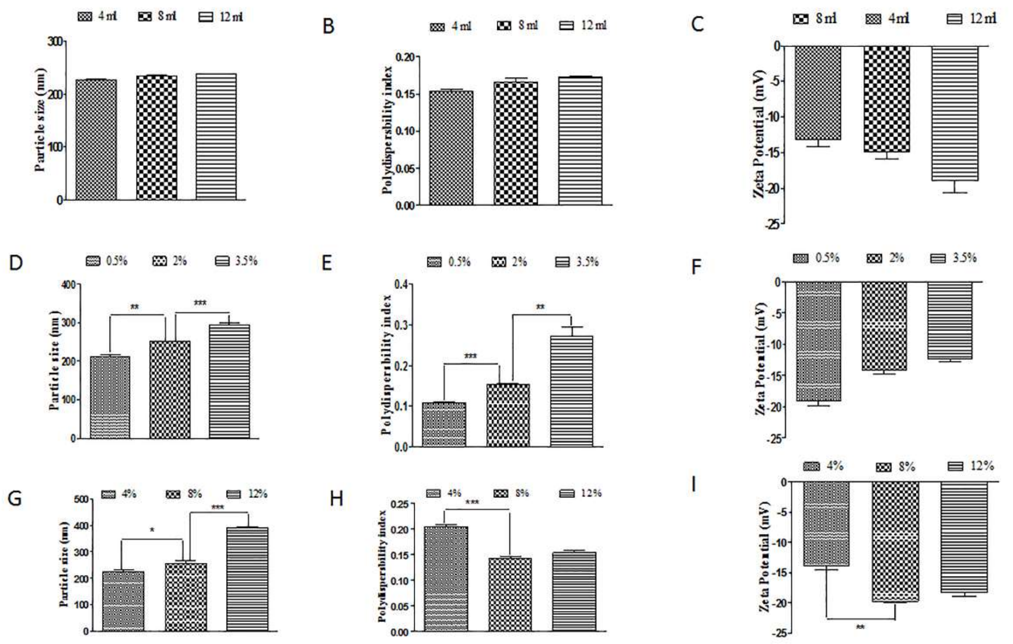

Figure S2. Effect of ethanol, albumin and glutaraldehyde concentrations on particle size, polydispersity index and zeta potential. The above figure shows the effect of ethanol amount on Particle size (**A**), polydispersity index (**B**) and zeta potential (**C**). It was reported that ethanol amount does not have any significant effect on particle size, polydispersity index and zeta potential. Values are mean ± SEM ($n = 3$). Effect of albumin concentration on particle size (**D**), polydispersity index (**E**) and zeta potential (**F**). It was demonstrated that increase in albumin concentration leads to increase in particles size (**D**) as well as the polydispersity index (**E**) significantly ($p < 0.001$). Polydispersity increases significantly when albumin concentration increases from 0.5% to 2%. However, it does not have any significant effect on zeta potential. Values are mean ± SEM ($n = 3$), ** $p<0.01$ and *** $p < 0.001$. Effect of increasing concentration of glutaraldehyde on particle size (G), polydispersity index (H) and zeta potential (**I**). It was evident from the graph that glutaraldehyde concentration increases particle size significantly ($p < 0.001$) when it increases from 4% to 12%. However, polydispersity index decreases significantly ($p < 0.001$) upon an increase in glutaraldehyde concentration but later it does not have any effect on PDI. Similarly, increase in glutaraldehyde concentration causes a significant ($p < 0.01$) decrease in zeta potential initially. Values are mean ± SEM (n = 3). * $p < 0.05$, ** $p < 0.01$, and *** $p < 0.001$.

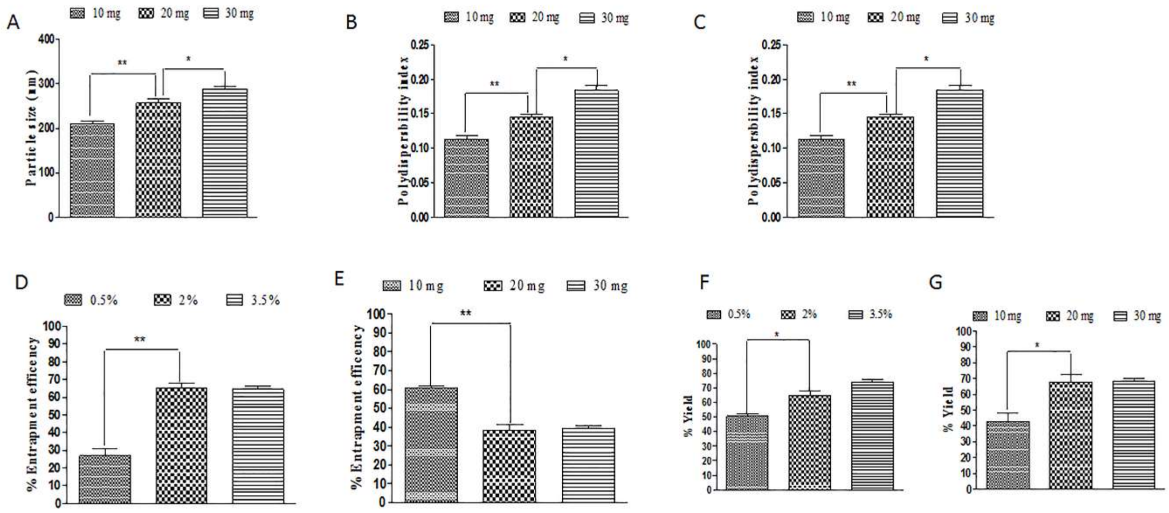

Figure S3. Effect of increase in drug amount on particle size, polydispersity index, zeta potential, albumin entrapment efficiency and percentage yield. The above Figure shows the effect of drug amount on Particle size (**A**), polydispersity index (**B**) and zeta potential (**C**). It is evident from the above graph that increases in PIC amount significantly ($p < 0.01$) increase the particle size and PDI. Similarly, zeta potential increases significantly ($p < 0.001$) upon an increase in PIC amount. Values are mean ± SEM ($n = 3$). * $p < 0.05$, ** $p < 0.01$ and *** $p < 0.001$.The figure S3 demonstrates effect of albumin concentration (**D**) and drug content (**E**) on entrapment efficiency. Figure S3D in the above graph show a significant difference ($p < 0.01$) when albumin concentration increases from 0.5% to 2%, however, entrapment efficiency slightly increases upon further increase in albumin concentration. Figure S3E in the above graph illustrates the significant decrease in entrapment efficiency ($p < 0.01$) as the drug amount increases from 10 mg to 20 mg but it does not have any significant effect when further increases to 30 mg. Values are mean ± SEM ($n = 3$). ** $p < 0.01$. Effect of albumin concentration (**F**) and drug amount (**G**) on percentage yield. Figure S3F in the above graph reveal a significant difference ($p < 0.05$) when albumin concentration increases from 0.5% to 2% although percentage yield slightly increases upon further increase in albumin concentration. Figure S3G in the above graph express no significant difference ($p < 0.05$) as the amount of drug increases. Values are mean ± SEM ($n = 3$). * $p < 0.05$.

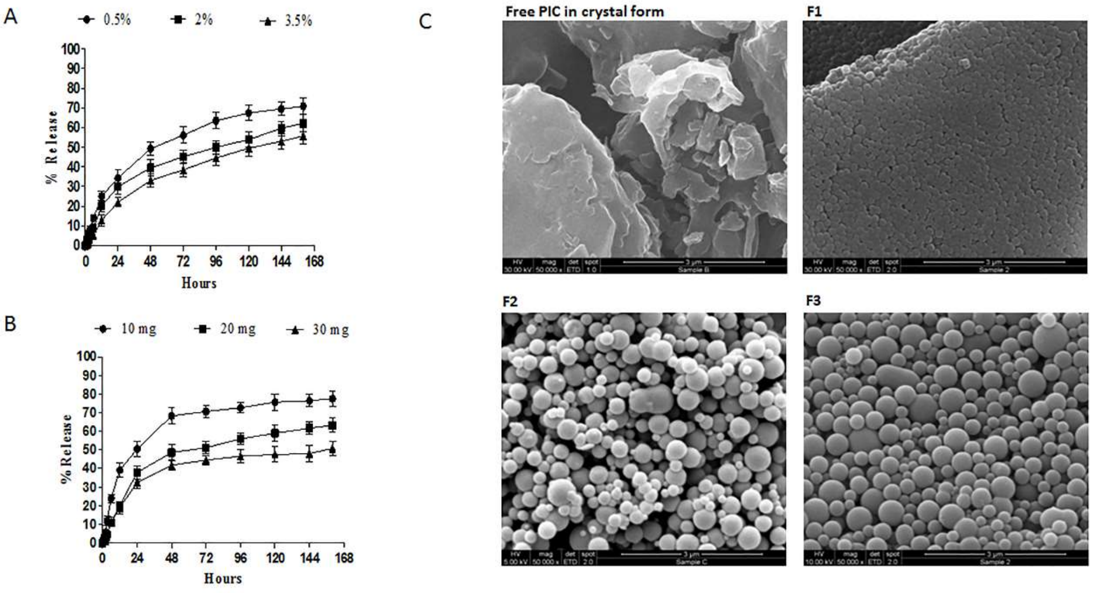

Figure S4. In vitro release and scanning electron microscopy of different batches of nanoparticles.Effect of albumin concentration (**A**) and drug amount (**B**) on In-vitro release profile. The above graph illustrates burst release after 24. The above graph depicts that increase in albumin and drug concentration leads to decrease the in-vitro of PIC release from the formulation. Values are mean ± SEM (n = 3). Figure S4C represents SEM imaging of free PIC, the batch containing 10 mg (**F1**), 20 mg (**F2**), and 30 mg (**F3**) of PIC used in the formulation. Batch formulated with 10 mg PIC represents smaller particles size whereas batch fabricated with 30 mg PIC shows larger particle size. F1 also demonstrate uniformity in size although F2 and F3 exhibit partial uniformity.

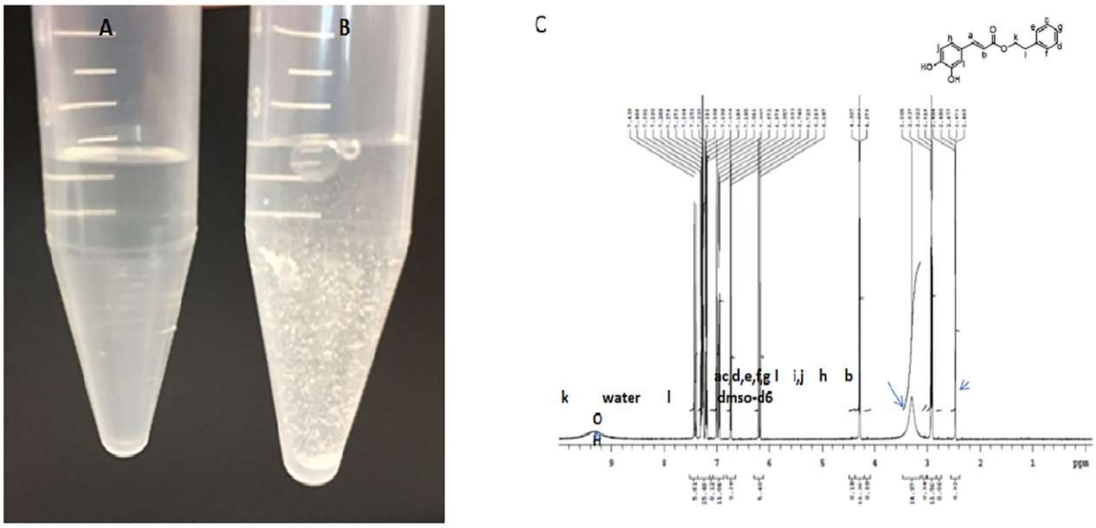

Figure S5. Solubility and NMR spectra of PIC-loaded nanoparticle. Water solubility of PIC-loaded albumin NP (**A**) and free PIC (**B**). The vials in (**A and B**) show the solubility of PIC-loaded albumin NP and free PIC, respectively. A Clear solution of PIC-loaded albumin NP in Figure A and turbid free PIC in Figure S5B can be observed above. Figure S5A indicates enhance in solubility of PIC-loaded albumin NP. Figure S5C shows H NMR of piceatannol pf albumin nanoparticles. The above H NMR confirms the piceatannol stucture in the nanoparticle form.

© 2020 by the authors. Licensee MDPI, Basel, Switzerland. This article is an open access article distributed under the terms and conditions of the Creative Commons Attribution (CC BY) license (http://creativecommons.org/licenses/by/4.0/).
